# Exploring the Interplay Between Early Maladaptive Schemas and Depression: A Comparative Analysis

**DOI:** 10.1192/j.eurpsy.2024.541

**Published:** 2024-08-27

**Authors:** A.-G. Zanfir

**Affiliations:** ^1^Prof. Dr. Alexandru Obregia Clinical Psychiatry Hospital; ^2^Faculty of Psychology and Educational Sciences, University of Bucharest, Bucharest, Romania

## Abstract

**Introduction:**

Depression, a pervasive mood disorder, significantly impairs one’s quality of life. Early Maladaptive Schemas (EMS), ingrained thought patterns stemming from early life experiences, play a pivotal role in shaping adult beliefs and behaviors. This study delves into the relevance of specific EMS domains—Emotional Inhibition (EI), Negativity/Pessimism (NP), and Social Isolation/Alienation (SI)—in influencing the severity of depression among medical students and diagnosed patients.

**Objectives:**

Our primary goal was to assess the correlation between specific EMS domains and depression severity in medical students and clinically diagnosed patients. We aimed to elucidate whether these schemas could serve as indicators for potential depressive tendencies or if they had a stronger association in those already diagnosed with depression.

**Methods:**

We conducted a prospective cross-sectional analysis involving 73 medical students and 61 diagnosed depression patients (aged 18-32). Four key variables—Depression, EI, NP, and SI—were measured using the Beck Depression Inventory-2 and The Young Schema Questionnaire-Short-form-3 in the Romanian context. Statistical analyses, including correlation coefficients and t-tests, were employed to explore the relationships between EMS domains and depression severity.

**Results:**

In the non-clinical sample, we identified moderate, statistically significant correlations between depression and EI (r=0.63), NP (r=0.71), and SI (r=0.59). Conversely, the clinical sample exhibited slightly weaker, yet significant correlations (EI-r=0.42, NP-r=0.39, SI-r=0.29). Notably, significant differences emerged between the groups in all measured variables. These findings imply that while a positive correlation between EMS variables and depression exists in both samples, the association weakens in diagnosed patients, indicating that these schemas may be less predictive in this population.

**Conclusions:**

Our study underscores the importance of understanding EMS domains in assessing depression severity. While specific schemas—EI, NP, and SI—correlate with depression in both medical students and diagnosed patients, this link is notably weaker in the latter group. Elevated EMS variables suggest a potential for future subclinical depression in medical students, but they might not strongly predict depression in those already diagnosed. These nuanced insights have implications for preventive interventions and therapeutic approaches tailored to individuals at different stages of depression, thereby enhancing targeted mental health care strategies.

**Disclosure of Interest:**

None Declared

